# Diet‐induced obesity in young mice: Consequences on the pancreatic intrinsic nervous system control of insulin secretion

**DOI:** 10.1002/edm2.95

**Published:** 2019-09-19

**Authors:** Marie‐Béatrice Saade, Armelle Cahu, Raphaël Moriez, Michel Neunlist, Sophie Blat

**Affiliations:** ^1^ Rennes Teaching Hospital, Pediatric and Clinical Genetic Center Rennes France; ^2^ INRA INSERM Univ Rennes NUMECAN Rennes France; ^3^ INSERM Univ Nantes IMAD TENS Nantes France

**Keywords:** endocrine pancreas, ex vivo model, Western diet

## Abstract

**Introduction:**

Obesity has become a pandaemic even in children. We aimed to investigate the impact of obesity in youth on later pancreatic intrinsic nervous system (PINS) phenotype and control of insulin secretion.

**Methods:**

Young mice (5‐week‐old, T0 group) were fed either a normal diet (ND group) or a Western diet (WD group) for 12 weeks. Pancreas nervous system density, PINS phenotype and pancreas anatomy were analysed by immunohistochemistry at T0 and in adulthood (ND and WD groups). Insulin secretion was also studied in these 3 groups using a new model of ex vivo pancreatic culture, where PINS was stimulated by nicotinic and nitrergic agonists with and without antagonists. Insulin was assayed in supernatants by ELISA.

**Results:**

Pancreas nervous system density decreased with age in ND (*P* < .01) but not in WD mice (*P* = .08). Western diet decreased the PINS nitrergic component as compared to normal diet (*P* < .01) but it did not modify its cholinergic component (*P* = .50). Nicotinic PINS stimulation induced greater insulin secretion in ND compared to WD mice (*P* < .001) whereas nitrergic stimulation significantly decreased insulin secretion in ND mice (*P* < .001) and tended to increase insulin secretion in WD mice (*P* = .08). Endocrine pancreas anatomy was not modified by the Western diet as compared to the normal diet (*P* = .93).

**Conclusions:**

Early Western diet induced neuronal density and phenotype changes in PINS that might be involved in the pancreas insulin secretion dysfunctions associated with obesity.

## Graphical abstract

Obesity starting in the young age has deep consequences on the maturation of the pancreas innervation (density and intrinsic nervous system phenotype) and the nervous control of insulin secretion. Western diet in childhood altered the normal course of pancreatic intrinsic nervous system maturation and impaired nicotinic control of insulin secretion.

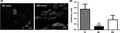

## 1 INTRODUCTION

Obesity has been declared a pandaemic by the World Health Organization and does not spare children.^1^ Obesity induces endocrine pancreatic dysfunction, such as impaired pulsatility^2^, ^3^ and loss of first phase insulin secretion,^4^ both under nervous control involving the pancreatic intrinsic nervous system (PINS).^5^, ^6^ PINS is made of perilobular ganglia and ganglia scattered within the parenchyma (intraparenchymal ganglia) sometimes in close proximity to the islets, whose terminal axons supply the endocrine and exocrine parts of the pancreas,^7^, ^8^ and new techniques of 3D panoramic histology with tissue clearing enabled to nicely visualize the neuro‐insular network in mice^9^ and humans.^10^ Although previous human islet imaging has suggested limited neural‐islet associations, particularly a lack of parasympathetic nerves in the islets,^11^, ^12^ these improved high‐definition images revealed that the sympathetic and parasympathetic nerves enter the islet core and are located in the immediate microenvironment of islet cells in humans as well as in mice.^9^, ^10^ PINS confers a functional autonomy to the pancreas as suggested by the spontaneous pulsatile insulin secretion displayed by ex vivo isolated pancreas 13 and could be responsible for the synchronization and the amplitude modulation of the pulses of insulin secretion.^5^, ^6^

Most PINS neurons are cholinergic^7^ and harbour nicotinic receptors.^14^ Their pharmacological activation stimulates insulin secretion both in vivo and in vitro.^15^, ^16^ Nitrergic neurons, which contain the neuronal isoform of the nitric oxide (NO) synthase enzyme (nNOS), are the second major subgroup.^17^ NO was reported to inhibit insulin release from isolated islets of mice in response to glucose and arginine,^18^ but to stimulate insulin secretion both in vivo and in vitro in various species including humans.^19^, ^20^, ^21^ Acetyl‐choline (ACh) and NO are often co‐expressed in pancreatic neurons.^22^

PINS’ role in the pathogenesis of the impaired insulin secretion in obesity is unclear. Yet, the cholinergic agonist carbachol has an increased insulinotropic activity in obese C57BL/6J mice compared to lean ones both in vivo^24^, ^25^ and in vitro.^26^ Islet hyperinnervation was found in a model of diet‐induced obesity in rats^23^ but not in mice.^25^ In type 2 diabetes mellitus (T2DM) Chinese hamsters, reduced islet innervation was reported,^27^ as was the remodelling of the mouse islet sympathetic innervation in diabetic injuries.^28^ To date, there is no data on how nitrergic neurons are involved but abnormalities in islet NO production have been reported in T2DM Goto‐Kakizaki rats^29^ and in islets of obese Zucker *fa/fa* rats and humans.^30^

Enteric nervous system (ENS) plasticity with early obesity has been described in mice, with gastric motility consequences.^31^ As ENS and PINS have the same embryologic origin and that PINS is usually considered as the pancreatic prolongation of ENS to which it is still connected by entero‐pancreatic neurons,^32^ we hypothesized that in addition to what occurs in ENS, early obesity could lead to plasticity of PINS, contributing to the alteration of insulin secretion. The maturation of the endocrine pancreas continues for several weeks postnatally, with a continuous increase in population size and discrete phases of renewed acceleration in growth activities, especially for beta cells.^33^ Studying the impact of obesity during this period of intense pancreatic development is therefore particularly relevant.^33^ We used a mouse model of early induction of obesity^31^ to characterize PINS plasticity and its functional consequences. In this well‐described model, mice display overweight at 7‐weeks of age, and increased weight, fat mass, leptinaemia and insulinaemia without change in fasting glycaemia but impaired glucose tolerance at 17 weeks of age.^31^

## 2 MATERIALS AND METHODS

### 2.1 Animals, diets and experimental protocol

Experimental protocol was approved by the Inserm Institutional Animal Care and Use Committee. It was part of a larger study whose results on the ENS and gastric motility have already been published.^31^ Male C57BL/6J Rj mice aged 4 weeks (Janvier Labs, Saint Berthevin, France) were maintained on a 12 hours light‐12 hours dark cycle (22°C) with free access to food and water. After 1 week of adaptation period, animals were randomly assigned to receive either a normal diet (ND group, Purified diet 210, SAFE, Augy, France) or a Western diet (WD group, Purified diet 230 HF, SAFE) for 12 weeks. At the completion of the experimental period, adult mice were killed by cervical dislocation. Five‐week‐old mice were used as initial controls (T0 group). Body and pancreas weights were recorded at sacrifice.

### 2.2 Immunohistochemical analysis

After fine dissection, pancreases were fixed in 4% paraformaldehyde for 3 hours either directly (endocrine pancreas anatomy and semi‐quantitative pancreas neurofilament density) or after stretching (PINS ganglia phenotype). Stretching consisted in pinning the pancreas flat in a dish lined with silicone (Sylgard^®^184; Sigma‐Aldrich, St Quentin Fallavier, France).^7^

#### 2.2.1 Endocrine pancreas anatomy and semi‐quantitative pancreas nervous system density

Pancreases (n = 5 per group) were processed for immunohistochemistry and analysed according to previously published protocols.^34^, ^35^, ^36^, ^37^ Cryosections were incubated overnight with primary antibodies (Table 1). After a washing step, sections were incubated for 3 hours with secondary antibodies (Table 1). Sections were rinsed again and mounted. Immunofluorescence was examined in epifluorescence microscope (Nikon EclipseE400; Nikon France S.A, Champigny‐sur‐Marne, France). Images were captured with a digital camera (Nikon Digital Still DXM1200; Nikon France S.A, France).

**Table 1:** Primary and secondary antibodies

Primary Antibodies			
Antigen	Host species	Dilution	Sources
Insulin	Guinea pig	1:200	Chemicon International; AB3440
Neurofilament H (NF)	Rabbit	1:500	Millipore; AB1989
Neuronal protein HuC/HuD (HU)	Mouse‐biotin	1:50	Invitrogen; A21272
Choline acetyltransferase (ChAT)	Goat	1:200	Millipore; AB144P
Neuronal nitric oxide synthase (nNOS)	Rabbit	1:1000	Enzo Life Sciences; ALX‐210‐501

Secondary Antibodies		
Antibody	Dilution	Sources
Rhodamine‐conjugated donkey anti‐guinea pig	1:100	Jackson ImmunoResearch; 706‐025‐148
Fluorescein‐conjugated donkey anti‐rabbit	1:200	Jackson ImmunoResearch; 711‐095‐152
DyLight^TM^488‐conjugated Streptavidin	1:200	Jackson ImmunoResearch; 016‐480‐084
Cy^TM^3‐conjugated donkey anti‐goat	1:500	Jackson ImmunoResearch; 705‐165‐003
Cy^TM^5‐conjugated donkey anti‐rabbit	1:500	Jackson ImmunoResearch; 711‐175‐152

Morphometry of the endocrine pancreas anatomy was performed as previously described.^35^ The whole section of the tissue was scanned using a digital slide scanner (Nanozoomer, Hamamatsu Photonics France SARL, Massy, France) and recorded files were analysed using ImageJ software (http://imagej.nih.gov/ij/) for automatic determination of the number and area of the islets within the section. Insulin‐positive area was calculated in each section (insulin‐positive area ratio to the total tissue area of the section). Islets were classified into five size categories (%) with respect to their diameter. Five evenly distributed sections were analysed and averaged per animal.

Pancreas nervous system density was assessed semi‐quantitatively as the relative frequency of neurofilament (NF)‐containing nerve fibres, as previously described for different pancreatic neurons.^25^ Briefly, the relative frequency of NF‐containing fibres was assessed semi‐quantitatively by the same observer on five evenly distributed sections per animal. The final score per animal was the mean of these five observations, which were graded as follows: 0: no NF‐immunoreactivity (IR), 1: occasional NF‐IR; 2: moderate NF‐IR; 3: numerous NF‐IR; 4: very abundant NF‐IR.

#### 2.2.2 PINS phenotype

After manual microdissection of pancreatic ganglia and attached connective tissues in the fixed stretched pancreases (n = 4‐8 per group), whole mounts were permeabilized for 1 hour in PBS‐Triton X‐100 (1%)‐horse serum (10%) and incubated overnight with primary antibodies (Table 1). After washing, whole mounts were incubated for 3 hours with secondary antibodies (Table 1). Preparations were mounted on glass slides and viewed under a fluorescence microscope (Nikon EclipseE400) attached to a digital camera (Nikon Digital Still DXM1200). Ganglia were spotted, photographed sequentially on their thickness and acquired with the NIS element software (Nikon France S.A). HU (pan‐neuronal marker)‐, NOS‐ and choline acetyltransferase (ChAT)‐IR neurons were counted in all the sequential pictures corresponding to one ganglion. At least 15 ganglia per mouse were studied to achieve a hundred studied neurons per animal.

### 2.3 Ex vivo measurement of intrinsic nervous control of insulin secretion

After cervical dislocation and laparotomy, the pancreas was finely excised and placed in ice‐cold Krebs solution (NaCl, 117 mmol/L; KCl, 4.7 mmol/L; MgCl_2_, 1.2 mmol/L; NaH_2_PO_4_, 1.2 mmol/L; NaHCO_3_, 25 mmol/L; CaCl_2_, 2.5 mmol/L and glucose, 11 mmol/L). It was transferred in an organ culture chamber containing 3 mL of Krebs solution warmed at 37°C and gassed with 95%O_2_ + 5%CO_2_. After a 30‐minutes equilibration period, Krebs solution was removed and replaced by 3 mL of fresh warmed one. As most PINS neurons harbour nicotinic receptors, PINS was stimulated using 1.1‐Dimethyl‐4‐Phenylpiperazinium iodide (DMPP, 100 µmol/L; Sigma‐Aldrich), a nicotinic agonist. The 100 µmol/L DMPP dose is classically used to stimulate the intrinsic nervous system (ENS).^38^ Supernatants (30 µL) were collected after 10, 20, 30, 45 and 60 minutes of stimulation and immediately frozen at −80°C for later analysis of insulin concentration.

#### 2.3.1 Validation of the ex vivo cultured pancreas viability and function

Viability and function were assessed by incubating the pancreases of mice aged 5 weeks with increasing concentrations of glucose (5.5, 11 and 25 mmol/L, n = 4‐5) for 60 minutes.

#### 2.3.2 Ex vivo stimulation of PINS and its effect on insulin secretion

DMPP (100 µmol/L)‐induced insulin secretion in preparations of pancreas from ND, WD and T0 mice (n = 5‐8) was measured. It was also measured in the presence of NOS inhibitor N^G^‐nitro‐l‐arginine methyl ester (L‐NAME, 50 µmol/L; Sigma‐Aldrich; n = 6) or NO donor sodium nitroprusside (SNP, 100 µmol/L; Sigma‐Aldrich, n = 5) to discriminate the role of PINS nitrergic component. L‐NAME was applied 20 minutes before DMPP stimulation.

#### 2.3.3 Assays

Insulin was assayed in supernatants using a specific ELISA kit for rat and mouse insulin (EZRMI‐13K; Millipore). The CV was 9.22% at a concentration of 28.5 μUI/mL and 5.34% at 92.3 μUI/mL. Incremental AUC was calculated on insulin secretion profiles by the trapezoidal method. Due to differences in absolute pancreas weight between groups, all the results of insulin secretion have been normalized to 100 mg of pancreas, to normalize between T0 (122 ± 4 mg), ND (156 ± 12 mg) and WD (199 ± 11 mg) mice pancreas weights.

### 2.4 Statistical analysis

Statistical analyses were performed with one‐way (diet/age) or two‐way (diet/age and PINS stimulation) ANOVA followed by Tukey's multiple‐comparison tests to determine statistical differences between T0, ND and WD mice as well as significant changes in insulin levels (areas under the curves) after different PINS stimulation within each group. The insulin profiles at different time points (0‐60 min) during the ex vivo challenges were compared using a two‐way repeated‐measures ANOVA (diet/age and time) to determine significant differences between T0, ND and WD mice. A Dixon test was primarily performed to remove out range values. As intended with Dixon test, one point maximum per data set was removed. Results are presented as means ± SEM. Differences were considered significant at *P* < .05.

## 3 RESULTS

### 3.1 Weight characterization

At sacrifice, WD mice were significantly heavier than ND mice (33.1 ± 0.8 g vs 24.8 ± 0.4 g for WD mice vs ND mice, *P* < .0001, n = 19‐20). WD mice pancreases were significantly heavier than those of ND mice (199 ± 11 mg vs 156 ± 12 mg for WD mice vs ND mice, *P* = .01, n = 19‐20) but not when considered in relative weight (relative to the mice body weight) (relative pancreas weight = 6.1 ± 0.4 mg/g vs 6.3 ± 0.5 mg/g for WD mice vs ND mice, *P* = .69, n = 19‐20).

### 3.2 Diet‐induced obesity modulated PINS phenotype

**Figure 1:**
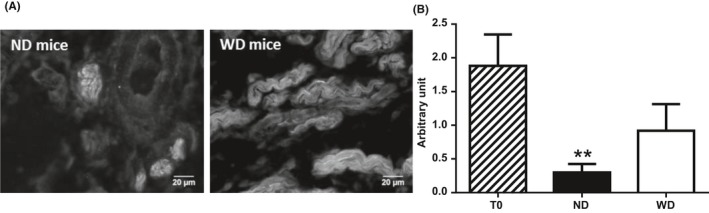
Pancreatic nervous system density semi‐quantitatively assessed as the relative frequency of neurofilament‐containing nerve fibres. A, immunohistochemical labelling of pancreatic neurofilaments in mice after 12 wk of normal diet (ND) or Western diet (WD). B, semi‐quantitative ranking of the relative frequency of neurofilament‐containing nerve fibres in 5‐wk‐old mice before dietary treatment (T0, n = 5), and in 17‐wk‐old ND (n = 5) and WD (n = 5) mice. Values are expressed as means ± SEM. **: *P* < .01 vs T0

Pancreas nervous system density, as semi‐quantitatively assessed, decreased significantly with age in ND mice (*P* < .01 between T0 and ND mice) but was not modified with age in WD mice (*P* = .08 between T0 and WD mice; Figure 1).

The number of neurons per ganglion was not significantly different between T0, ND and WD mice (6.37 ± 0.81, 4.25 ± 1.10 and 4.72 ± 0.71 neurons per ganglion in T0, ND and WD mice respectively, *P* = .33).

**Figure 2:**
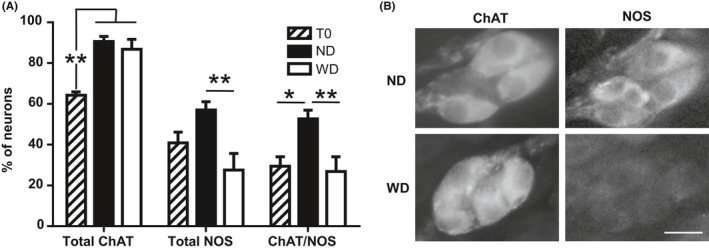
A, Percentage of neurons immunoreactive for choline acetyltransferase (total ChAT) and nitric oxide synthase (total NOS) or both (ChAT/NOS) before (T0, n = 4) and after 12 wk of a normal diet (ND, n = 6) or a Western diet (WD, n = 8) in mice. B, typical immunohistochemical staining of pancreatic ganglia in ND and WD mice. **: *P* < .01, *: *P* < .05. Bar = 15 µmol/L

The total proportion of cholinergic neurons increased significantly with age in both ND and WD mice (*P* < .01) and was not significantly different between ND and WD mice (*P* = .50; Figure 2). Inversely, the total proportion of nitrergic neurons was significantly lower in WD mice compared to ND mice (*P* < .01) due to an opposite evolution of this population of neurons with age in WD compared to ND mice. The proportion of neurons displaying both ACh and NO increased with age in ND mice (*P* = .04) but not in WD mice (*P* = .79) resulting in a significant difference between ND and WD mice (*P* < .01; Figure 2).

### 3.3 Diet‐induced obesity decreased PINS stimulation of insulin secretion

#### 3.3.1 Validation of the ex vivo model

**Figure 3:**
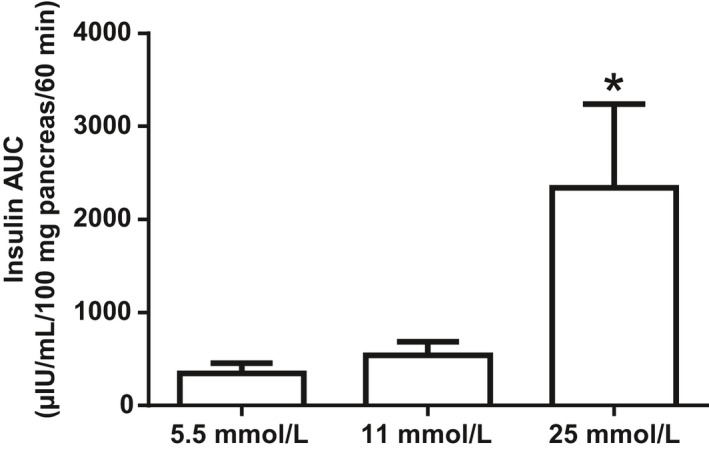
Insulin secretion (AUC, μIU/mL/100mg pancreas/60min) of ex vivo cultured pancreas of 5‐week‐old mice under glucose stimulus (5.5, 11 and 25 mmol/L). *, *P* < .05, n = 4‐5

Cultured pancreases of T0 mice secreted significantly higher amount of insulin when stimulated with glucose 25 mmol/L compared to glucose 5.5 (*P* = .03) and 11 mmol/L (*P* = .04) (Figure 3). The 11 mmol/L glucose concentration was chosen for the subsequent experiments of PINS stimulation as this concentration of glucose did not stimulate insulin secretion per se and allowed better energy supply to the non‐perfused pancreases.

#### 3.3.2 Ex vivo study

**Figure 4:**
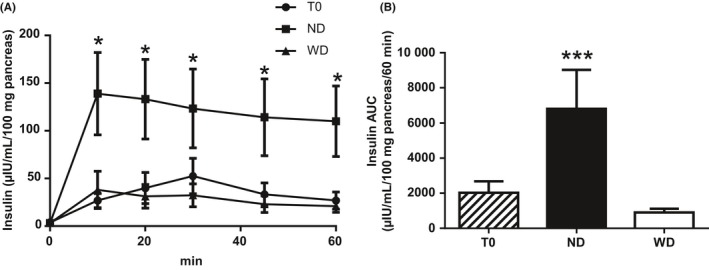
Insulin secretion profile (A) and AUC (B) (μIU/mL/100mg pancreas/60min) of ex vivo cultured pancreas of mice before (T0, black circles, n = 8) and after 12 wk of a normal diet (ND, black squares, n = 5) or a Western diet (WD, black triangles, n = 5) under pancreatic intrinsic nervous system stimulation (DMPP, Dimethylpiperazinium, nicotinic agonist, 100 µmol/L). Means ± SEM. *, *P* < .05; ***, *P* < .001

PINS stimulation by 100 µmol/L DMPP induced a significantly higher insulin secretion in ND compared to WD mice and T0 mice (*P* < .001; Figure 4). DMPP‐induced insulin secretion in WD mice resembled that of T0 mice (*P* = .32) (Figure 4).

**Figure 5:**
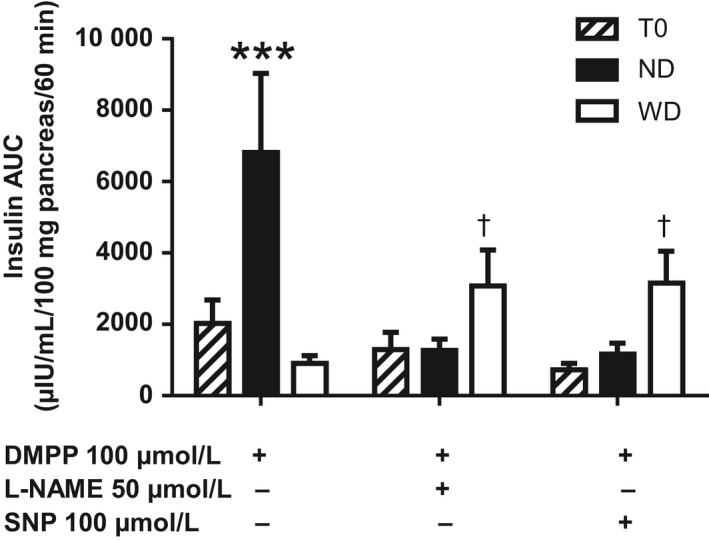
Insulin secretion (AUC, µIU/mL/100 mg pancreas/60 min) of ex vivo cultured pancreas of mice before (T0, n = 6‐8) and after 12 wk of a normal diet (ND, n = 4‐5) or a Western diet (WD, n = 5‐6) under pancreatic intrinsic nervous system stimulation (DMPP, Dimethylpiperazinium, nicotinic agonist, 100 µmol/L) in the presence of the nitric oxide synthase inhibitor N^G^‐nitro‐l‐arginine methyl ester (L‐NAME, 50 µmol/L) or in the presence of the NO donor sodium nitroprusside (SNP, 100 µmol/L). Means ± SEM. ***, *P* < .001; †, *P* < .1 vs WD DMPP 100 µmol/L

Addition of L‐NAME 50 µmol/L, a nitrergic blocker, significantly inhibited insulin release in ND mice, as did SNP 100 µmol/L, the NO donor (*P* < .001), while L‐NAME and SNP tended to increase insulin secretion in WD mice as compared to DMPP alone (*P* = .07 and *P* = .08 for L‐NAME and SNP respectively; Figure 5). Addition of L‐NAME or SNP had no effect on DMPP‐stimulated insulin secretion in T0 mice (*P* = .49 and 0.25 for L‐NAME and SNP respectively; Figure 5).

### 3.4 Diet‐induced obesity did not modify endocrine pancreas maturation

**Figure 6:**
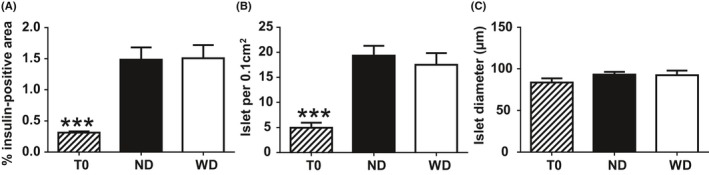
Percentage of insulin‐positive area (A), number of islets/0.1 cm^2^ (B) and mean islet diameter (µm, C) in pancreases of mice before (T0, n = 5) and after 12 wk of a normal diet (ND, n = 5) or a Western diet (WD, n = 5). Means ± SEM. ***, *P* < .001

WD did not alter the maturation of the endocrine pancreas as the percentage of endocrine tissue significantly increased in both WD and ND mice compared to T0 mice (*P* < .001; Figure 6). The number of islets per 0.1 cm^2^ of tissue was significantly greater in ND and WD mice as compared to T0 (*P* < .001), while the mean islet size was not modified regardless of age or mice diet (Figure 6). There was no change in the distribution of islets, regardless of age or diet (data not shown).

## 4 DISCUSSION

The objective of this work was to characterize the effects of diet‐induced obesity starting in childhood on the later PINS anatomy and control of pancreatic endocrine function in a well‐described mouse model of diet‐induced obesity, characterized by hyperinsulinaemia and impaired glucose tolerance.^31^ We used a new model of ex vivo pancreas culture, in which we demonstrated a dose‐dependent insulin secretion in response to glucose consistent with a preserved pancreatic function. The functional study to assess the PINS role was conducted at the whole pancreas scale. We found that obesity starting in the young age has deep consequences on the maturation of the pancreas innervation (density and PINS phenotype) and the nervous control of insulin secretion. WD altered the normal course of PINS maturation and impaired nicotinic control of insulin secretion.

We used a semi‐quantitative approach targeting NF to evaluate the pancreas neuronal density. We found that pancreas neuronal density was lower in ND mice compared to T0 showing a natural decrease with ageing. In contrast, WD induced preservation of pancreas nervous system density with ageing. These results were consistent with Baudry et al^31^ who demonstrated the same preservation of the stomach ENS density with WD. However, despite differences in NF density, the number of neurons per ganglion was not different between T0, ND and WD mice in contrary to what was described in the ENS.^31^ Cholinergic neurons proportion increased significantly with age in both ND and WD groups but the nitrergic component was significantly decreased in WD mice compared to ND. The proportion of neurons co‐expressing ACh and NO was significantly lower in WD mice as compared to ND mice and resembled that of T0 mice. These results suggested that WD altered the normal course of PINS maturation. The mechanisms of such an alteration have been approached in the mice ENS, where an increase in glial‐derived neurotrophic factor (GDNF) in obese animals has been described due to their higher leptinaemia.^31^ Similar mechanism could occur in the PINS, which is the prolongation of the ENS at the pancreatic level and in which glial cells have been described.^7^, ^32^ Thus, the higher pancreas nervous system density observed in obese mice did not imply the cholinergic and nitrergic neurons but may imply other pancreatic neurons such as vasoactive intestinal polypeptide and neuropeptide Y neurons as already described in islets of diet‐induced obese rats,^23^ but not mice.^25^ Sympathetic innervation may also be implied since islet sympathetic hyperinnervation (×2) has been demonstrated in an experimental diabetes model in mice.^28^ However, these latter studies focused on islet innervation and did not consider the pancreas in its entirety.

We assessed the functional consequences of pancreas nervous system density and phenotype modifications with the Western diet in a new model of ex vivo pancreas culture. This model was validated in mice aged 5 weeks. We demonstrated a dose‐dependent insulin secretion when pancreases were stimulated by glucose 25 mmol/L as compared to glucose 5.5 and 11 mmol/L (*P* < .05), consistent with a preserved pancreatic function. The small release of insulin induced by glucose 5.5 mmol/L validated the tissue integrity with no massive release of insulin by lysed cells. We further confirmed the tissue integrity by analysing by IHC the endocrine pancreas anatomy on two 60 minutes‐cultured pancreases (data not shown).

The functional study to assess the PINS role was conducted at the whole pancreas scale. We used the nicotinic receptor agonist DMPP to perform a global ganglionic activation as almost all PINS neurons harbour nicotinic receptors.^14^ DMPP stimulation of PINS induced major insulin secretion in ND mice but induced only a weak insulin secretion in WD mice, similar to that of T0 mice and suggesting an impaired PINS sensitivity to nicotinic stimulation at the ganglia level in WD mice. The islet hypersensitivity to cholinergic stimulus described in high‐fat diet treated C57BL/6J^24^ may therefore be compensatory for this impaired sensitivity at the ganglia level.

Addition of the NO donor SNP induced a significant decrease in DMPP‐induced insulin secretion in ND mice whereas it tended to increase insulin secretion in WD mice. The inhibitory action of NO on insulin secretion in ND mice is in accordance with some of the existing literature.^18^ The tendency of NO donors to enhance insulin release in WD mice highlighted their PINS alteration and may pass through a stimulation of insulinotropic neurons or an inhibition of inhibitory neurons.^39^

In ND mice, addition of L‐NAME, a NOS inhibitor, inhibited DMPP‐induced insulin secretion. This was quite surprising since NO inhibited insulin secretion in our model. We used a low dose of L‐NAME (50 µmol/L) in order to target the nNOS present in the peripheral ganglia, and not the one on the beta cells.^40^ A higher dose (5 mmol/L) displayed similar results (results not shown). One explanation of this result could be that L‐NAME directly inhibited beta‐cell insulin release due to its muscarinic receptor blocking activity.^41^ For further studies, it would be interesting to use more specific nNOS inhibitors such as N^G^‐methyl‐l‐arginine (L‐MMA), an endogenous inhibitor of nNOS derived from in vivo proteolysis of methylated arginine residues on various proteins^42^ or N^G^‐nitro‐l‐arginine (L‐NNA), a competitive inhibitor of NOS with selectivity for the neuronal and endothelial isoforms of the enzyme, which don't have this muscarinic receptor blocking activity. In WD mice, L‐NAME tended to increase insulin secretion highlighting the deleterious effect of early obesity on nervous insulin control secretion.

The anatomy of the endocrine pancreas (% of insulin‐positive areas, number and size of islets) was not different between ND and WD mice but both groups had an endocrine tissue percentage significantly greater than 5‐week‐old mice, in accordance with previous data in rats.^33^ This was due to an increase in the islet number, with no change in islet size, as already described in mice and humans.^43^ The absence of islet hypertrophy in early stages of obesity was already described in adults rats (at 2, 4 and 8 weeks of high‐fat diet)^23^ and in the same type of mice after 12 weeks of diet.^25^ Beta‐cell hyperplasia was described in obese mice but after 12 months of WD.^44^ In our model, mice were hyperinsulinaemic with normal fasting glycaemia but impaired glucose response to an OGTT after 12 weeks of WD.^31^ After 12 weeks of WD, mice were in a prediabetes state with no hypertrophy of the endocrine pancreas yet. The pancreas was still able to secrete enough insulin to counteract the insulin resistance, due to a higher activation of the insulin biosynthetic machinery and an increased insulin turnover as already described in the same model of obese mice.^25^ PINS plasticity would therefore be the earliest phenomenon occurring in the pathogenesis leading from obesity to type 2 diabetes.

In conclusion, we demonstrated that obesity starting in the young age had deep consequences on the maturation of the PINS with later functional consequences on nervous control of insulin secretion in adult mice, which could lead to the development of type 2 diabetes. The mechanisms of this plasticity and the main neuronal populations involved remain to be determined.

## CONFLICT OF INTEREST

The authors declare no conflict of interest.

## AUTHOR CONTRIBUTIONS

MN, RM and SB conceived the project and designed the experiment. MBS, RM and SB performed the experiments and MBS, AC and SB performed the analysis. MBS and SB analysed the data. MBS and SB wrote the draft manuscript. All authors reviewed and approved the final manuscript.

## ETHICAL APPROVAL

The present study was designed and conducted in compliance with the current ethical standards of the European and French guidelines (directive 2010/63/EU and decree 2013‐118, respectively). The Inserm Institutional Animal Care and Use Committee approved the entire protocol. Animals were observed daily throughout the experimental protocol to ensure their welfare and they received no medication or antibiotic treatment.

## Data Availability

The data sets generated during the current study are available from the corresponding author on reasonable request.
